# Effects of payer status on breast cancer survival: a retrospective study

**DOI:** 10.1186/s12885-015-1228-7

**Published:** 2015-04-01

**Authors:** Runhua Shi, Hannah Taylor, Jerry McLarty, Lihong Liu, Glenn Mills, Gary Burton

**Affiliations:** 1 Department of Medicine & Feist-Weiller Cancer Center, LSU Health Shreveport, 1501 Kings Hwy, Shreveport, LA 71103 USA; 2 Feist-Weiller Cancer Center, LSU Health Shreveport, 1501 Kings Hwy, Shreveport, LA 71103 USA

**Keywords:** Female breast cancer, Survival, Payer status, Insurance, Risk factors

## Abstract

**Background:**

Breast cancer outcomes are influenced by multiple factors including access to care, and payer status is a recognized barrier to treatment access. To further define the influence of payer status on outcome, the National Cancer Data Base data from 1998–2006 was analyzed.

**Method:**

Data was analyzed from 976,178 female patients diagnosed with breast cancer registered in the National Cancer Data Base. Overall survival was the primary outcome variable while payer status was the primary predictor variable. Secondary predictor variables included stage, age, race, Charlson Comorbidity index, income, education, distance travelled, cancer program, diagnosing/treating facility, and treatment delay. Multivariate Cox regression was used to investigate the effect of payer status on overall survival while adjusting for secondary predictive factors.

**Results:**

Uninsured (28.68%) and Medicaid (28.0%) patients had a higher percentage of patients presenting with stage III and stage IV cancer at diagnosis. In multivariate analysis, after adjusting for secondary predictor variables, payer status was a statistically significant predictor of survival. Patients with private, unknown, or Medicare status showed a decreased risk of dying compared to uninsured, with a decrease of 36%, 22%, and 15% respectively. However, Medicaid patients had an increased risk of 11% compared to uninsured. The direct adjusted median overall survival was 14.92, 14.76, 14.56, 13.64, and 12.84 years for payer status of private, unknown, Medicare, uninsured, and Medicaid respectively.

**Conclusion:**

We observed that patients with no insurance or Medicaid were most likely to be diagnosed at stage III and IV. Payer status showed a statistically significant relationship with overall survival. This remained true after adjusting for other predictive factors. Patients with no insurance or Medicaid had higher mortality.

## Background

In 2014, there will be an estimated 232,670 new cases of breast cancer and approximately 40,000 deaths in the United States [1]. The estimated prevalence for women living with breast cancer in the United States was 3,131,440 [2]. The median age of diagnosis for breast cancer was 61 years [2]. The age-adjusted breast cancer incidence rate for women was 124.6 per 100,000 [3]. While the age-adjusted incidence rate was similar between white and black women, black women had higher mortality than white women [4].

Payer status, as well as income, education, age, and ethnicity, may affect access to health care and influence breast cancer stage at diagnosis [5] and patient survival [5-9]. Reduced access to healthcare has been linked to advanced stage of cancer [5,7] and worse survival [6,7]. Lower survival rates have been found in individuals with no insurance or Medicaid [6,7,10,11]. Lower education attained has been associated with large tumor size and advanced stage disease at breast cancer diagnosis [12], however, the association with patient survival has been mixed [13,14].

With the recent development of the Affordable Care Act [15], there may be a shift in health insurance coverage in the US. In the 2012 population, there were 50.90 million (16.4%) people enrolled in Medicaid, 48.88 million (15.7%) with Medicare, and 47.95 million (15.4%) with no insurance [16]. As the type and availability of insurance changes, it will be important to assess differential effects of payer status on the outcome of patient survival. This study used the large National Cancer Data Base (NCDB) data to evaluate how payer status, as well as secondary factors, impacts breast cancer survival.

Secondary factors, which may also reflect access to healthcare, include the following indicators: (1) The patient’s choice of treatment facility type (cancer program), (2) whether they are diagnosed and treated in the same facility (diagnosing/treating facility), (3) the distance a patient must travel to the facility (distance travelled), (4) the length of the delay to start treatment once diagnosed (treatment delay), and (5) their Charlson Comorbidity index.

Studies have demonstrated an improved prognosis for female breast cancer patients treated in large community hospitals compared with small community hospitals and Health Maintenance Organization (HMO) hospitals [17]. This is supported by evidence that shows better outcomes for high-risk surgery in high-volume hospitals [18]. Teaching hospitals, known for awareness of current treatment methods and higher medical research involvement, have also shown an advantage over nonteaching facilities [17,19,20]. Stage at diagnosis has been linked to distance travelled for healthcare [21]. Differences in survival rates [22] and timely mammography for breast cancer in women [23] have been found between urban and rural settings. A few studies have found that treatment delay has no significant relationship with breast cancer survival [24-26]. In contrast, one study found an 85% increased risk of breast cancer-specific mortality for low-income, late-stage breast cancer patients who waited >60 days to initiate treatment compared to those who waited <60 days [27]. More co-existing conditions or a higher Charlson Comorbidity index has also been found to be a predictor of late stage diagnosis in colon cancer [21] and to be associated with increased risk of breast cancer mortality [28]. This study investigated the effects of payer status on female breast cancer survival.

## Method

This study examined 976,178 female breast cancer patients who were diagnosed between 1998 and 2006 and followed until December 31, 2011. The data used in this study was derived from a de-identified NCDB file. The NCDB captures approximately 70% of all newly diagnosed cases of cancer in the United States at the institutional level [29]. The International Classification of Disease for Oncology, third edition (ICD-O-3) codes (C500-C506, and C508, C509) associated with a diagnosis of breast cancer were used to select patients.

The primary outcome variable, survival time of breast cancer patients, was calculated from date of diagnosis to date of death, date of loss to follow-up, or date of study end (December 31, 2011). The primary predictor variable was payer status. Secondary predictor variables included tumor stage, age, race, Charlson Comorbidity score, income, education, distance travelled, cancer program, diagnosing/treating facility, and treatment delay.

Payer status was categorized as uninsured, private, Medicaid, Medicare (or other government insurance plan), or unknown. The American Joint Committee on Cancer (AJCC) stage was categorized as I, II, III, or IV for stage at diagnosis. Age was grouped as 18–49, 50–64, 65–74, or ≥75 years. Patient race was categorized as white, black, or other. The other race category included patients with Asian and Hispanic ethnicity. Charlson Comorbidity [28] is an index to reflect the overall health status of a patient. Charlson Comorbidity was categorized as 0, 1, ≥2, or unknown. Income, or median household income at zip code level, was grouped as < $30, $30-34, $35-45, or ≥ $46 k. Education, a measure of the percent of adults in the patient's zip code who did not graduate from high school, was grouped as ≥29%, 20-28%, 14-19%, and <14%. Education was determined using 2000 census data. Distance travelled, the distance from the patient’s residential zip code to a medical center, was grouped as <10, 10–24, 25–49, 50–99, or ≥100 miles. Cancer program was categorized as community, comprehensive, academic and research, or other (other services and clinics) cancer program. Diagnosing/treating facility was categorized as same or different. Treatment Delay was grouped as 0–5, 6–20, 21–30, or ≥31 days.

Chi-Square statistical tests were used to compare the distributions of stage by payer status and other categorical variables. Kaplan-Meier methods were used to estimate survival curves. Log rank tests were used to compare the survival distributions in univariate analysis. Šidák correction method was used for adjustment in Multiple Comparisons for the Log rank Test. Multivariate Cox regression was used to simultaneously estimate the hazard of death (Hazard Ratio) of payer status and adjusted other factors. Direct Adjusted Median Overall Survival (MOS) was calculated by using Multivariate Cox regression. Statistical Software SAS 9.4 (SAS Inc. Gary, NC) and STATA 13.1 (College Station, TX: Stata Corp LP) were used for data management, statistical analysis, and modeling. All p-values <0.05 were considered statistically significant.

## Results

The mean age at diagnosis for all patients was 60 years, with mean ages of 60.5, 56.5, and 54.8 years for white, black, and other race respectively. The mean age at diagnosis was 61.5, 58.2, 58.1, and 61.8 years for stage I, II, III, and IV respectively.

The patient’s payer status distribution by stage is shown in Table 1. For stage, 47.96%, 37.04%, 10.37%, and 4.62% of patients presented with stage I, II, III, and IV diseases, respectively. For payer status, 2.55%, 55.61%, 4.27%, 34.23%, and 3.34% of patients presented with uninsured, private, Medicaid, Medicare, and unknown payer status at diagnosis, respectively. Uninsured (28.68%) and Medicaid (28.0%) patients had a much higher proportion of advanced stage (stage III and IV) disease. Private (13.28%) and Medicare (12.79%) had a lower proportion of stage III and stage IV disease. A statistically significant difference in the presentation of advanced stage at diagnosis was found according to payer status (p < 0.05).

**Table 1:** **Insurance payer status distribution by stage of female breast cancer patients**

Stage		Uninsured	Private	Medicaid	Medicare	Unknown	Total
I	n	7464	259536	12546	174552	14124	468222
	%	30.04	47.81	30.07	52.23	43.29	47.96
II	n	10260	211217	17492	110231	12418	361618
	%	41.29	38.91	41.93	32.99	38.06	37.04
III	n	4281	53672	7826	31570	3911	101260
	%	17.23	9.89	18.76	9.45	11.99	10.37
IV	n	2844	18380	3854	17829	2171	45078
	%	11.45	3.39	9.24	5.34	6.65	4.62
III + IV	n	7125	72052	11680	49399	6082	146338
	%	28.68	13.28	28	14.79	18.64	14.99
Total	n	24849	542805	41718	334182	32624	976178
	%	2.55	55.61	4.27	34.23	3.34	100

A statistically significant association was also found between stage at diagnosis and all secondary factors (data not shown). African American patients (28.15%) had the highest stage III and stage IV, and the percentages for white (14.06%) and other (14.47%) were much lower. Distinct patterns appeared in the stage distributions of Charlson Comorbidity, income, and education. As the Charlson Comorbidity increased, the percentage of stage II, III, and IV patients increased. As income and education level increased, the percentage of stage II, III, and IV patients decreased.

The results of univariate analysis can be seen in Table 2. For payer status, the MOS value for each level was statistically different from all other levels. Medicare payer status had the shortest MOS (MOS = 10.13 years), followed by Medicaid (13.08), unknown (14.56), uninsured (>14.89), and private (15.00).

**Table 2:** Median overall survival (MOS)* for female breast cancer patients

	Factor	Level	n	MOS	Lower	Upper
Group factors	All Patients		976178	14.75	14.69	14.84
Demographic	Age (Years)	18-49	252080	>14.99	N/A	N/A
		50-64	354102	>14.99	14.94	N/A
		65-74	192042	13.59	13.48	13.73
		≥75	177954	7.14	7.1	7.18
	Race	White	851863	14.77	14.71	14.86
		Black	98641	13.25	13.03	13.66
		Others	25674	>14.95	N/A	N/A
	Education (%), did not graduate from high school	≥29	144440	13.5	13.35	13.74
		20-28	202583	14.09	13.95	14.16
		14-19	217587	14.49	14.37	14.64
		<14	366118	14.92	14.9	N/A
Clinical characteristic	Stage	Stage I	468222	14.99	14.92	N/A
		Stage II	361618	14.78	14.69	14.94
		Stage III	101260	7.81	7.7	7.91
		Stage IV	45078	1.7	1.67	1.73
	Charlson Comorbidity	0	375270	10.33	10.16	N/A
		1	41661	9.6	9.45	9.85
		≥2	8322	5.58	5.4	5.81
		Unknown	550925	14.71	14.67	14.8
Access to health care	Payer Status	Uninsured	24849	>14.89	14.06	N/A
		Private	542805	15	N/A	N/A
		Medicaid	41718	13.08	12.84	13.85
		Medicare	334182	10.13	10.08	10.18
		Unknown	32624	14.56	14.31	N/A
	Income ($1000)	<30	117919	12.77	12.65	12.92
		30-34	158762	13.92	13.79	14.05
		35-45	256013	14.47	14.33	14.51
		≥46	398086	14.92	14.9	N/A
	Distance Travelled (Miles)	<10	556476	14.58	14.47	14.66
		10-24	234049	15	N/A	N/A
		25-49	88994	14.77	14.63	N/A
		50-99	39053	>14.94	14.64	N/A
		≥100	22349	14.99	N/A	N/A
	Cancer Program	Community	110174	13.72	13.56	13.83
		Comprehensive	563299	14.75	14.69	14.86
		Academic Research	261484	>14.99	14.9	N/A
		Others	41221	12.49	12.19	13.12
	Diagnosing/Treating Facility	Same	662498	14.61	14.5	14.67
		Different	313680	14.91	14.86	N/A
	Treatment Delay (Days)	0-5	296904	14.76	14.69	14.92
		6-20	284321	14.86	14.77	N/A
		21-30	155774	14.84	14.67	N/A
		≥31	193455	14.45	14.24	14.7

*All p-values <0.0001 by using Logrank Test. Median Overall Survival (MOS). N/A: not reached.

Overall MOS was 14.75 years. With the exception of distance travelled and treatment delay, all secondary factors showed an MOS value for each level that was statistically different from all other levels. The largest differences were found for stage, age, and Charlson Comorbidity. MOS decreased as stage, age, and Charlson Comorbidity increased. Age ≥75 (7.14) and ≥2 Charlson Comorbidity (5.58) had the shortest survival for their groups. Stage III and IV (1.70) had much shorter survival compared to stage I and II. Education and income displayed a more subtle pattern. As the patient’s level of education and income increased, MOS also increased.

MOS was statistically inferior for distance travelled greater than 50 miles. Results for MOS according to treatment delay did not follow a clear pattern. Patients with treatment delay of 0–5 days and ≥31 days were not statistically different from each other but differed from the other delay groups (6–20 and 20–30 days).

Tables 1 and 2 demonstrate the need for multivariate regression to further investigate the effect of payer status. In these analyses, many factors are statistically related to survival.

Table 3 displays the results of hazard ratio (HR) of death from a multivariate cox regression analysis. After adjusting for secondary factors, payer status was a significant predicator for overall survival. Private, unknown, and Medicare payer status had a decreased risk of dying compared to uninsured, with decreases of 36% (HR = 0.64), 22% (0.78), and 15% (0.85) respectively. Patients with Medicaid insurance, however, had an 11% (1.11) increased risk of dying as compared to uninsured patients had.

**Table 3:** Hazard ratio (HR) of death and 95% confidence interval (CI) of HR* from multivariate Cox regression analysis for female breast cancer patients

			Hazard ratio, 95% CI			
Group	Factor	Level	HR	Lower	Upper	p-value^#^
Demographic	Age (Years)	18-49	1			
		50-64	1.12	1.1	1.13	<.0001
		65-74	1.66	1.63	1.69	<.0001
		≥75	4.00	3.93	4.07	<.0001
	Race	White	1			
		Black	1.31	1.29	1.33	<.0001
		Others	0.78	0.75	0.8	<.0001
	Education (%), did not graduate from high school	≥29	1			
		20-28	1	0.98	1.01	0.6894
		14-19	0.96	0.95	0.98	<.0001
		<14	0.89	0.88	0.91	<.0001
Clinical characteristic	Stage	Stage I	1			
		Stage II	1.82	1.8	1.84	<.0001
		Stage III	4.5	4.45	4.56	<.0001
		Stage IV	15.54	15.32	15.75	<.0001
	Charlson Comorbidity	0	1			
		1	1.43	1.41	1.46	<.0001
		≥2	2.27	2.19	2.34	<.0001
		Unknown	1.24	1.22	1.25	<.0001
Access to health care	Payer Status	Uninsured	1			
		Private	0.64	0.62	0.65	<.0001
		Medicaid	1.11	1.08	1.15	<.0001
		Medicare	0.85	0.82	0.87	<.0001
		Unknown	0.78	0.76	0.81	<.0001
	Income ($1000)	<30	1			
		30-34	0.96	0.95	0.98	<.0001
		35-45	0.95	0.94	0.96	<.0001
		≥46	0.89	0.88	0.91	<.0001
	Distance Travelled (Miles)	<10	1			
		10-24	0.97	0.96	0.98	<.0001
		25-49	0.95	0.94	0.96	<.0001
		50-99	0.93	0.91	0.95	<.0001
		≥100	0.9	0.87	0.93	<.0001
	Cancer Program	Community	1			
		Comprehensive	0.95	0.94	0.96	<.0001
		Academic Research	0.90	0.89	0.92	<.0001
		Others	1.08	1.05	1.11	<.0001
	Diagnosing/Treating Facility	Same	1			
		Different	0.91	0.9	0.92	<.0001
	Treatment Delay (Days)	0-5	1			
		6-20	0.93	0.92	0.94	<.0001
		21-30	0.90	0.89	0.92	<.0001
		≥31	0.98	0.96	0.99	<.0001

*HR: Hazard Ratio of death. CI: Confidence Interval.

#p-value: Chi-test of HR is significantly different from 1 (the reference group of each factor).

For example, HR = 0.64 (0.62-0.65) for private payer status indicated that, adjusting for stage, age, race, etc. the patient with private payer status has a 36% (1–0.64 = 0.36) lower risk of dying compared to uninsured payer status.

Adjusting for other factors, age, race, Charlson Comorbidity index, and stage were also significant predictors of survival in Table 3. HR increased with increasing age. The HR was higher for age 50–64 (1.12), 65–74 (1.66), and ≥75 (4.0) compared with age 18–49. At age ≥75, patients were 4.0 times more likely to die than those age 18–49. Compared to white patients, black patients had a 31% (1.31) increase, and other race had a 22% (0.78) decrease. Patients with ≥2 (2.27) and 1 (1.43) Charlson Comorbidity were more likely to die than those with no comorbid conditions. Corresponding to the subtle pattern in Table 2, HR decreased as both income and education increased.

**Figure 1:**
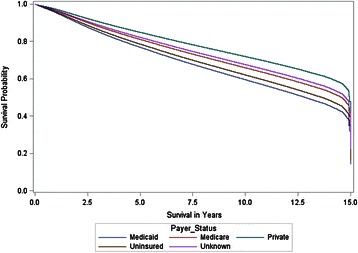
**Direct adjusted survivor functions for payer status.** Direct adjusted median overall survival (MOS) was 14.9, 14.8, 14.6, 13.6, and 12.8 years for private, unknown, Medicare, uninsured, and Medicaid respectively.

Figure 1 illustrates the Direct Adjusted MOS found for payer status only. The Direct Adjusted MOS was 14.92, 14.76, 14.56, 13.64, and 12.84 years for private, unknown, Medicare, uninsured, and Medicaid payer status respectively. Patients with private insurance had a 2.1 year longer survival compared to patients with Medicaid insurance.

## Discussion

Payer status had a statistically significant relationship to stage distribution and overall survival for female breast cancer patients. Patients with no insurance or Medicaid had the highest proportion at stage III and stage IV when diagnosed (Table 1), a finding which supports the results of another study [5]. In multivariate analysis, adjusting for other factors including stage, payer status was a significant predictor of overall survival. Patients with private and unknown payer status were less likely to die than uninsured and Medicaid patients. Private patients had a Direct Adjusted MOS over 1.3 and 2.1 years longer than uninsured and Medicaid patients respectively (Figure 1). Our findings support other studies that show higher stage at diagnosis [5] and worse survival for patients with no insurance or Medicaid [6,7,10,11]. Higher stage at diagnosis and lower survival in these populations might be explained by lower access to preventive screening and high-quality care. Further research is needed to investigate the barriers for these populations and to develop targeted interventions.

In the multivariate analysis, the secondary significant predictors of survival were age, race, Charlson Comorbidity index, and stage. Patient’s age ≥75 were 4.00 times more likely to die than patients 18–49. As expected, older patients have a higher risk because of the aging process. African American patients had the highest mortality when compared to white patients. This was consistent with literature demonstrating lower survival in African American patients [9,30,31]. Patients with ≥2 Charlson Comorbidity were 2.27 times more likely to die than those with no comorbid conditions. Another study indicated an association of one unit of change of Charlson Index with a 2.3-fold increase in the 10-year mortality in breast cancer patients [28]. As a measure of overall health status, a higher risk of dying is expected with a higher Charlson Index.

In this study, the HR estimation for various factors was more reliable, with a narrow 95% confidence interval, because so many patients were studied. However, because of this, the reader must differentiate between statistical and clinical significance when interpreting the results. Although all categories in the multivariate analysis were statistically significant, not all HR changes would be clinically important. For example, with an HR of 0.96, some factors were statistically significant even though there was only a risk reduction of 4%.

This study investigated how a patient’s access to health care can impact survival. The level of patient adherence to National Comprehensive Cancer Network treatment guidelines was not studied here, but could also be an important factor. Addressing patient adherence in future research might provide a more complete understanding of the influence of treatment characteristics.

Another issue was the information collected from the NCDB. The database did not collect Charlson Comorbidity information consistently before 2003. The reference group (0 Charlson Comorbidity) for 2003–2006 was used to estimate the Charlson Comorbidity effect for patients diagnosed before 2003 (coded as unknown Charlson Comorbidity). This estimate may only represent an average of all Charlson Comorbidity conditions in the earlier group. The NCDB also did not collect cause-specific death information. We assessed the effect of payer status on overall survival instead of cause-specific survival. Measuring the effect on cause-specific survival may produce different results. Additionally, education and income by zip-code was collected instead of individual education and income. Using individual education and income level would strengthen the analysis of these factors. The NCDB, a large retrospective national database, may also be sensitive to bias in patient selection and variation in institution reporting [32].

## Conclusion

We observed that uninsured and Medicaid patients were most likely to be diagnosed at stage III and stage IV. Payer status, our primary focus, showed a statistically significant relationship with overall survival. This remained true after adjusting for secondary predictive factors. Patients with no insurance or Medicaid had higher mortality than private, Medicare, unknown insurance. Further research is needed to investigate patient treatment adherence and cause-specific survival.

### Ethics statement

With the support from the Chair of Louisiana State University Hospital in Shreveport (currently University Health Shreveport) Cancer program, the corresponding author has applied and has been awarded the National Cancer Data Base (NCDB) Participant Use Data File (PUF) for 1998 to 2011 from the Commission on Cancer (CoC). The PUF is a Health Insurance Portability and Accountability Act (HIPAA) compliant data file containing cases submitted to the Commission on Cancer’s (CoC) National Cancer Data Base (NCDB). The PUF contains de-identified patient level data that do not identify hospitals, healthcare providers, or patients as agreed to in the Business Associate Agreement that each CoC-accredited program has signed with the American College of Surgeons. The PUFs are designed to provide investigators associated with CoC-accredited cancer programs with a data resource they can use to review and advance the quality of care delivered to cancer patients through analyses of cases reported to the NCDB. NCDB PUFs are only available through an application process to investigators associated with CoC-accredited cancer programs.

## Abbreviations

HMO: Health Maintenance Organization
NCDB: National Cancer Data Base
AJCC: American Joint Committee on Cancer
MOS: Median overall survival
HR: Hazard ratio
